# Self-reported hypertension prevalence, risk factors, and knowledge among South Africans aged 24 to 40 years old

**DOI:** 10.1038/s41371-024-00957-8

**Published:** 2025-02-24

**Authors:** Asanda Mtintsilana, Witness Mapanga, Ashleigh Craig, Siphiwe N. Dlamini, Shane A. Norris

**Affiliations:** 1 DSI-NRF Centre of Excellence in Human Development, University of the Witwatersrand, Johannesburg, Gauteng South Africa; 2 SA MRC/Wits Developmental Pathways for Health Research Unit, Department of Paediatrics, Faculty of Health Sciences, School of Clinical Medicine, University of the Witwatersrand, Johannesburg, South Africa; 3 School of Physiology, Faculty of Health Sciences, University of the Witwatersrand, Johannesburg, South Africa; 4 School of Human Development and Health, University of Southampton, Southampton, UK

**Keywords:** Lifestyle modification, Hypertension

## Abstract

Although hypertension is a significant public health burden in South Africa (SA), less is known about its prevalence, risk factors, and possible preventative strategies among young adults. We assessed the prevalence, possible risk factors, and knowledge associated with self-reported hypertension among young adults from SA. A cross-sectional online survey was conducted among 1000 young South African adults (24–40 years; 51.0% women). We administered a socio-demographic questionnaire and collected information on measures of socio-economic status (SES) (e.g. asset wealth index), self-reported medical history, and lifestyle risk factors. Furthermore, a modified version of the hypertension evaluation of lifestyle and management questionnaire was used to assess participants’ hypertension knowledge. The overall prevalence of self-reported hypertension was 24.0%, with significant differences between women and men (27.5% and 20.4% respectively, p = 0.033). Only 16.8% of the respondents had good hypertension knowledge. There was a positive association between good knowledge of hypertension and being hypertensive (OR = 1.43 CI:1.23–3.12), monthly blood pressure check-ups (OR = 2.03 CI:1.78–3.23), knowing the side effects of uncontrolled blood pressure (OR = 1.28 CI:1.07–1.89) and having a biological mother with hypertension (OR = 1.79 CI:1.53–2.21). Being employed full-time (OR = 0.74 CI:0.69–0.80), having a higher SES (wealth index 4 (OR = 0.70 CI:0.59–0.97) and 5 (OR = 0.65 CI:0.48–0.81)), exercising 6 to 7 days per week (OR = 0.83 CI:0.71–0.94), and not consuming alcohol at all (OR = 0.73 CI:0.67–0.89), were all found to be protective against hypertension. The high hypertension prevalence, lack of hypertension knowledge, and reported risk factors among this group highlight the need for early robust preventative strategies to mitigate hypertension risk among this population.

## Introduction

Approximately 1.3 billion adults between the ages of 30 and 79 years have arterial hypertension, defined as systolic blood pressure (SBP) ≥ 140 mmHg and/or diastolic blood pressure (DBP) ≥ 90 mmHg (140/90 mmHg), or taking treatment for hypertension [1]. Sub-Saharan Africa (SSA) has the highest reported prevalence of hypertension (27%) and countries such as South Africa (SA) are disproportionally affected [1, 2]. For example, a multi-country study performed in six sites from four countries including South Africa (SA) showed that the three South African sites (Soweto, Agincourt, and Dikgale) had the highest proportion of hypertensive adults (54.1%, 47.3%, and 41.5% respectively) [2]. Regrettably, the prevalence of childhood hypertension is also higher in the SSA region (6.9%) compared to the global prevalence (4.0%) [3].

Worrisome is that several studies conducted in SA have provided substantial evidence showing that hypertension and elevated BP (defined as SBP/DBP of 130–139 mmHg/80–85 mmHg) developed in childhood and adolescence track into adulthood [4–6]. Additionally, a recent study by Wandai et al. [7] showed that BP levels may increase between 30 and 60 years of age, which suggests that the implementation of preventative strategies during this window period may assist in combating the high and increasing prevalence of hypertension in SA. This is crucial as raised BP levels during this period are associated with increased peripheral vascular resistance and large arterial stiffness, which are prominent risk factors for developing cardiovascular diseases (CVDs) such as heart failure and stroke [8, 9]. Collectively, these findings might partly explain why four out of ten leading causes of mortality in SA in 2016 were hypertension and related diseases such as 'other forms of heart disease' and cerebrovascular diseases [10]. These diseases accounted for 17.4% of deaths in 2016 [10].

The increase in blood pressure levels or in the prevalence of hypertension with age has been mostly associated with structural changes in large artery stiffness, which subsequently leads to high pulse pressure and pulse wave velocity [8, 9, 11]. Several biological mechanisms underlying hypertension include metabolic syndrome or components of metabolic syndrome, inflammation and vascular endothelial dysfunction, and oxidative stress [8, 9, 11]. For example, age-related increases in body fat, visceral adipose tissue, and circulating leptin have been associated with abnormal adipokine production and an increase in inflammatory cytokines and chemokines. These inflammatory responses may cause vascular endothelial dysfunction and arterial stiffening, and hypertension-associated organ damage [8, 9, 11]. Similarly, hyperglycaemia and dyslipidemia, components of metabolic syndrome, have been implicated in vascular endothelial dysfunction and oxidative stress, resulting in vascular remodelling and arterial stiffening.

The evidence that a person’s disease status is influenced early in life, suggests that it is essential to establish preventative strategies earlier [4–6]. This notion aligns closely with the Developmental Origins of the Health and Disease (DOHaD) paradigm [12, 13]. This paradigm highlights the importance of applying a life-course epidemiological approach in understanding how socio-environmental exposures during early development or other critical window periods of vulnerability can lead to adverse health outcomes throughout the life courses and can even perpetuate this cycle into future generations [12, 13]. This approach is paramount in tackling the high and increasing prevalence of hypertension in SA or the SSA region, where there is a dearth of prevention and treatment strategies for hypertension [2, 14–16]. This, in part, explains the low levels of awareness among African patients surrounding their hypertension status and poor BP control, and inadequate health knowledge about the potential benefits of treatment and prevention strategies [2, 14–16]. These barriers may also explain why SA has a high prevalence of hypertension-related risk factors such as overweight or obesity and unfavourable lifestyle behaviours such as excessive smoking, and alcohol intake [17, 18].

Therefore, there is a need for continuous monitoring of hypertension prevalence, knowledge, and awareness among vulnerable groups, which include children, adolescents, and young adults. Therefore, this study aimed to investigate the prevalence of hypertension, possible risk factors, and related hypertension knowledge among 24–40-year-old South African men and women. Specifically, 24–40-year-olds were selected as the age group representing an important period for enhancing preventative strategies to alleviate the rising prevalence of hypertension and potentially delay the onset of CVDs in the South African population. This is crucial as this age group is often associated with worse hypertension awareness, treatment, and control compared to their middle-aged and older adult counterparts [14, 19].

## Methods

### Survey integrity and processes

**Fig. 1:**
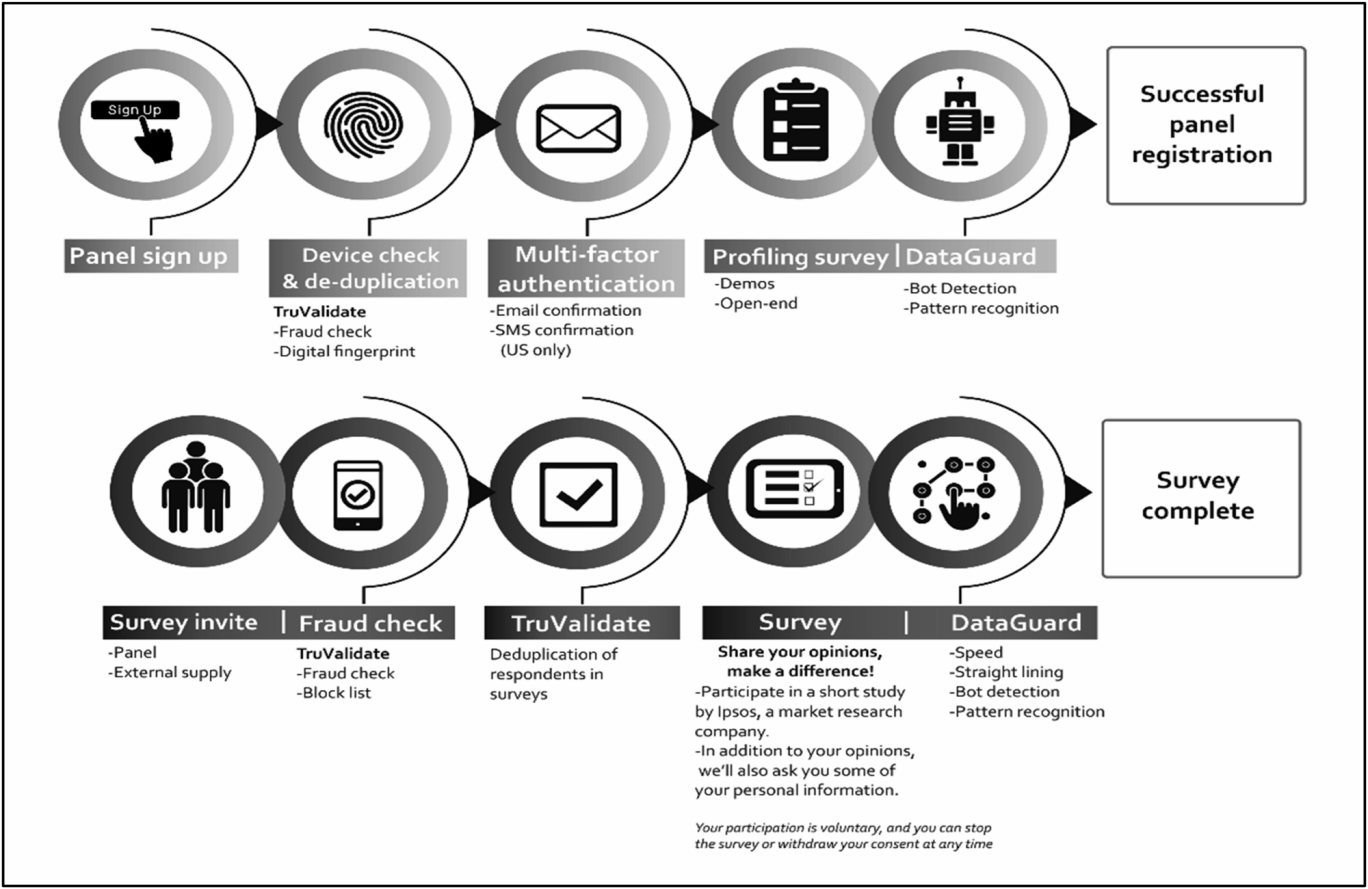
Institut Public de Sondage d’Opinion Secteur (IPSOS) i-Say respondent registration process and survey completion [18].

This online cross-sectional survey was conducted in July 2022 using an 'i-Say panel' as shown in Fig. 1. The 'i-Say panel' has been described in our previous study using a multi-country cohort [20]. Briefly, prospective panellists (i.e. respondents) were recruited across the nine provinces of SA via a multisource recruitment process that involved the use of digital fingerprinting technology (TruValidate) to collect the respondent’s device information. Other processes included fraud checks, deduplication reviews, and the detection of invalid responses that included copied and pasted text, unrealistic typing speed, and giving the same answer to all questions in a grid [20]. Respondents with suspicious activity were removed in real time. The survey ended when 1000 valid respondents completed the survey, and their responses were verified using the various anti-fraud checks that are described above.

Notably, we only included respondents between 24 and 40 years of age (50.0% female) with internet access. Therefore, the recruited sample may not necessarily represent the general population of SA.

### Data collection

An online demographic questionnaire written in English was administered through IPSOS in the respondent’s language of choice (e.g. English, IsiXhosa, IsiZulu, Setswana, and Tshivenda) and included demographic factors and measures of socio-economic status (SES) such as age, gender, ethnicity, household assets, housing density, employment, and education status. A wealth index score was calculated from a list of 12 household amenities (i.e. smartphone/mobile phone, computer or tablet, television, refrigerator, vehicle, washing machine, microwave, flush toilet, tap water, electricity, generator, and air conditioner) and used as a measure of SES. Previous studies have used this score in this setting [21, 22].

Self-reported medical history information was obtained by asking the respondents if they had been told by a medical doctor or any other health worker if they have any of these diseases: hypertension/high BP, high cholesterol, diabetes, overweight or obesity, chronic kidney disease, and/or common mental health conditions such as anxiety and depression. Moreover, current smoking status and frequency were recorded and categorised into the following four groups: (i) 0–1; (ii)1–5; (iii) 6–10; and (iv) 11 or more. Notably, smoking was not only limited to cigarettes but also included other forms of tobacco use such as chewing tobacco, pipe, cigars, vape, hubbly, and snuff or dagga. Similarly, alcohol intake and frequency were measured and categorised in the following groups: occasionally/not every week; once a week; 2–3 times a week; and every day. Furthermore, physical activity was recorded as the average number of days in a week the respondent engaged in physical activity (>30 min/day) that was hard enough to increase their heart rate or break a sweat.

Respondents were also asked if they had a first- and second-degree relative with a history of hypertension/high BP, high cholesterol, diabetes, overweight or obesity, heart disease/heart attack, stroke, and/or mental health conditions. Furthermore, an updated hypertension evaluation of lifestyle and management (HELM) questionnaire with 22 multiple-choice questions was administered [23, 24] (Supplementary Table S1, questions 10 to 32). The HELM questionnaire was modified to include 'yes, no, or don’t know' responses, and questions on current hypertension knowledge, definition/diagnosis, treatment or control, prevention of hypertension, and lifestyle changes for hypertension prevention [23, 24]. The questionnaire also included another question about the respondents’ confidence in detecting and treating hypertension. The responses to the questionnaire were scored by assigning 1 point for each correct or 'positive' (e.g. 'Yes' to knowing what low-sodium salt) answer out of the 22 questions. Participants were classified as having less knowledge if they scored below 7, intermediate knowledge with a score between 7 and 12, and good knowledge if they scored more than 12. Moreover, respondents were asked questions about their beliefs and perceptions of ten potential strategies to prevent, reduce, and manage hypertension (Supplementary Table S1, questions 33 to 50).

### Statistical analyses

All statistical analyses were performed using Stata® version 17 (Stata Corp Ltd, College Station, Texas, USA). The general characteristics of the study respondents were analysed using descriptive statistics. Chi-square tests (χ^2^) with confidence intervals were used to determine the association between the binary scores and the independent variables of interest (socio-demographic characteristics, lifestyle factors, province, general health, and disease burden). We analysed the socioeconomic and demographic, hypertension knowledge, behaviours, and general health and disease burden factors of the respondents by sex. We used the Pearson chi-squared test to compare the distributions of values of categorical variables and by sex. We then conducted a hierarchical multivariate logistic regression analysis to examine the associations between hypertension knowledge, behaviours, socioeconomic and demographic factors, and being hypertensive. This approach enabled us to investigate complex hierarchical multi-level relationships among the various factors. Model 1 of the hierarchical regression assessed the association between hypertension knowledge and being hypertensive, while model 2 assessed behaviours (smoking, drinking alcohol, physical activity, and sleeping patterns) and being hypertensive. All the variables (hypertension knowledge, behaviours, socioeconomic and demographic factors) were added in model 3 to determine their association with hypertension. Odds ratios were used to determine the strength of association between the various factors and hypertension. All the analyses were two-sided, and the p-value was highly significant at <0.001 and moderately significant at <0.05.

### Ethical approval statement

Since IPSOS is a market research house and member of research institutions, it does not have to obtain ethical approval for their panels. Also, panel surveys are opt-in surveys, meaning respondents choose to become a member understanding the data privacy policy and confidentiality procedures. IPSOS complies to the industry standards and regulations set out by institutions including the South African Market Research Association, Protection of Personal Information Act in South Africa, and the European Society for Opinion and Market Research (ESOMAR). Kenya also adheres to ESOMAR and the UK adheres to the Market Research Society and General Data Protection Regulation regulations.

Furthermore, we obtained ethical clearance from the University of Witwatersrand Human Ethics Research Committee (Non-Medical) (ethics clearance certificate number: H21/06/36). All methods were performed in accordance with the relevant guidelines and regulations or declaration of Helsinki.

All eligible members of the panel population were sent an invitation to participate and the information sheet about the study. Informed consent was obtained from all the respondents who participated and completed the survey. The participants were informed that privacy and anonymity would be maintained.

## Results

### Descriptive statistics

Table 1 shows the study population characteristics stratified by sex (N = 1000; 51.0% women). Most of the respondents in the study reside in the Gauteng (47.6%), Western Cape (14.8%), or KwaZulu-Natal (13.60%) provinces. In terms of the highest level of education attained, there were no sex differences evident, with the majority of the respondents having a tertiary/university/college level education (59.6% of women vs 53.5% of men).

**Table 1:** Descriptive statistics of socio-demographic and lifestyle factors stratified by sex.

Variables	Total N(%)	Women n(%)	Men n(%)	p-value
	1000 (100.00)	510 (51.00)	490 (49.00)	
Province/region				
Eastern Cape	62 (6.2)	33 (6.5)	29 (5.9)	0.146
Free State	38 (3.8)	14 (2.7)	24 (4.9)	
Gauteng	476 (47.6)	232 (45.5)	244 (49.8)	
KwaZulu-Natal	136 (13.6)	75 (14.7)	61 (12.4)	
Limpopo	47 (4.7)	20 (3.9)	27 (5.5)	
Mpumalanga	50 (5.0)	23 (4.5)	27 (5.5)	
North West	32 (3.2)	19 (3.7)	13 (2.6)	
Northern Cape	11 (1.1)	6 (1.2)	5 (1.0)	
Western Cape	148 (14.8)	88 (17.2)	60 (12.2)	
Highest level of education				
Completed grade 12 (have a high school certificate)	392 (39.2)	190 (37.2)	202 (41.2)	0.065
Primary school/some high school	42 (4.2)	16 (3.1)	26 (5.3)	
Tertiary/university/college degree	566 (56.6)	304 (59.6)	262 (53.5)	
Current employment status				
Employed fulltime	573 (57.3)	292 (57.2)	281 (57.3)	0.773
Employed parttime	196 (19.6)	95 (18.6)	101 (20.6)	
Studying	53 (5.3)	27 (5.3)	26 (5.3)	
Unemployed	178 (17.8)	96 (18.8)	82 (16.7)	
Smoking cigarettes/vape				
No	520 (52.0)	329 (64.5)	191 (39.0)	**<0.001**^**a**^
Yes	480 (48.0)	181 (35.5)	299 (61.0)	
Consuming alcohol				
2–3 times per week	203 (20.3)	79 (15.5)	124 (25.3)	**<0.001**^**a**^
Every day	27 (2.7)	11 (2.2)	16 (3.3)	
Not at all	160 (16.0)	110 (21.6)	50 (10.2)	
Occasionally/not	431 (43.1)	247 (48.4)	184 (37.5)	
Once per week	179 (17.9)	63 (12.3)	116 (23.7)	
Wealth index				
1 (poorest)	200 (20.0)	84 (16.5)	116 (23.7)	**0.024**^**a**^
2	200 (20.0)	103 (20.2)	97 (19.8)	
3	200 (20.0)	113 (22.2)	87 (17.8)	
4	200 (20.0)	112 (22.0)	88 (18.0)	
5 (wealthiest)	200 (20.0)	98 (19.2)	102 (20.8)	

Categorical data are presented as frequencies, n(%).

^a^This is a significant result.

Overall, 48.0% of the respondents in the study sample reported smoking cigarettes/vaping (n = 480) and 61.0% of these were men, and 20.3% consume alcohol 2–3 times per week, with men drinking more than women. There were differences in wealth status (determined by SES) between women and men, with women more in the middle strata of the statuses.

### General health and burden of disease

A total of 24.0% of the respondents reported hypertension, with significant differences between women and men. The prevalence of hypertension among women was 27.5% as compared to 20.4% among men. Also, there were differences in common mental health conditions between women and men, with women more likely to report a mental health condition than men, 28.4% vs 17.8% (Table 2).

**Table 2:** General health status, the burden of disease, and knowledge of hypertension stratified by sex.

Variables	Total N(%)	Women n(%)	Men n(%)	p-value
	1 000 (100.0)	510 (51.0)	490 (49.0)	
The self-reported burden of disease				
Hypertension	240 (24.0)	140 (27.5)	100 (20.4)	**0.033**^**a**^
Hypercholesterolaemia/dyslipidaemia	121 (12.1)	60 (11.8)	61 (12.4)	0.68
Diabetes	111 (11.1)	56 (11.0)	55 (11.2)	0.76
Obesity/overweight	202 (20.2)	117 (22.9)	85 (17.3)	0.084
Chronic kidney disease	52 (5.2)	23 (4.5)	29 (5.9)	0.60
Mental health conditions	232 (23.2)	145 (28.4)	87 (17.8)	**<0.001**^**a**^
The average number of days you exercise per week				
0	119 (11.9)	63 (12.4)	56 (11.4)	0.798
1	116 (11.6)	55 (10.8)	61 (12.5)	
2	182 (18.2)	94 (18.4)	88 (18.0)	
3	243 (24.3)	129 (25.3)	114 (23.3)	
4	135 (13.5)	72 (14.1)	63 (12.9)	
5	145 (14.5)	72 (14.1)	73 (14.9)	
6	28 (2.8)	11 (2.2)	17 (3.5)	
7	32 (3.2)	14 (2.8)	18 (3.7)	
Your ability to manage stress				
Average	366 (36.6)	193 (37.8)	173 (35.3)	**0.028**^**a**^
Fair	145 (14.5)	79 (15.5)	66 (13.5)	
Good	301 (30.1)	151 (29.6)	150 (30.6)	
Poor	57 (5.7)	36 (7.1)	21 (4.3)	
Very good/excellent	131 (13.1)	51 (10.0)	80 (16.3)	
Your quality of sleep				
Poor	123 (12.3)	57 (11.2)	66 (13.5)	0.760
Fair	173 (17.3)	93 (18.2)	80 (16.3)	
Average	285 (28.5)	144 (28.2)	141 (28.8)	
Good	310 (31.0)	161 (31.6)	149 (30.4)	
Very good/excellent	109 (10.9)	55 (10.8)	54 (11.0)	
Knowledge of hypertension				
Less knowledge	82 (8.2)	35 (6.9)	47 (9.6)	0.290
Intermediate knowledge	750 (75.0)	388 (76.1)	362 (73.9)	
Good knowledge	168 (16.8)	87 (17.0)	81 (16.5)	
How often is your blood pressure checked?				
Monthly	187 (18.7)	117 (22.9)	70 (14.3)	**<0.001**^**a**^
Quarterly	172 (17.2)	88 (17.3)	84 (17.1)	
Bi-annually	243 (24.3)	132 (25.9)	111 (22.7)	
Annually	216 (21.6)	84 (16.5)	132 (26.9)	
Never	182 (18.2)	89 (17.5)	93 (19.0)	
Known side effects of uncontrolled blood pressure				
No	313 (31.3)	131 (25.7)	182 (37.1)	**<0.001**^**a**^
Yes	591 (59.1)	326 (63.9)	265 (54.1)	
Do not know	96 (9.6)	53 (10.4)	43 (8.8)	
Known hypertension				
Biological mother	348 (34.8)	199 (39.0)	149 (30.41)	**0.004**^**a**^
Biological father	205 (20.5)	98 (19.2)	107 (21.8)	0.305
Biological grandparent	275 (27.5)	138 (27.1)	137 (28.0)	0.750
Biological sibling (brother/sister)	72 (7.2)	36 (7.1)	36 (7.4)	0.860
Known high blood cholesterol or fat				
Biological mother	99 (9.9)	56 (11.0)	43 (8.8)	0.243
Biological father	75 (7.5)	35 (6.9)	40 (8.2)	0.435
Biological grandparent	100 (10.0)	43 (8.4)	57 (11.6)	0.092
Biological sibling (brother/sister)	40 (4.0)	22 (4.3)	18 (3.7)	0.606
Known diabetic				
Biological mother	150 (15.0)	76 (14.9)	74 (15.1)	0.929
Biological father	148 (14.8)	83 (16.3)	65 (13.3)	0.180
Biological grandparent	257 (25.7)	142 (27.8)	115 (23.5)	0.114
Biological sibling (brother/sister)	35 (3.5)	19 (3.7)	16 (3.3)	0.692

Categorical data are presented as frequencies, n(%).

^a^This is a significant result.

Regardless of the significant differences in hypertension prevalence among women and men, there were no differences evident when it came to respondents’ knowledge of hypertension, with the majority of respondents having intermediate knowledge with a score between 7 and 12 out of a total of 19. There were major differences between women and men in the understanding of known side effects of uncontrolled BP, with women likely to know more than men. Furthermore, there were no significant differences between women and men when it comes to their parents, siblings, or grandparents having other associated diseases such as diabetes or high cholesterol.

### Determinants of hypertension status

Before carrying out the hierarchical multiple logistic regression models, the relationship between knowledge of hypertension and behaviours (smoking, drinking alcohol, and managing stress) was carried out and there were no apparent associations between the variables. As Table 3 shows, the increase in hypertension knowledge indicated a likelihood of being hypertensive, there was no association between having knowledge of hypertension and being hypertensive (model 1). In model 2, those who do not consume alcohol and those who occasionally consume alcohol were 35.0% and 24.0% less likely to be hypertensive, respectively, when compared to those who consume alcohol two to three times a week. Furthermore, those who did physical exercises for 4–5 days and 6–7 days, were 10.0% and 32.0% less likely to be hypertensive, respectively, when compared to those who did not exercise at all.

**Table 3:** Hierarchical multiple logistic regression models of the associations between hypertension knowledge, behaviours, socioeconomic and demographic factors, and being hypertensive among the 24–40 age group in South Africa.

Variables	Model 1 (hypertension knowledge vs hypertension status) OR (95% CI)	Model 2 (behaviours vs hypertension status) OR^2^ (95% CI)	Model 3 (hypertension knowledge, behaviours, and socioeconomic and demographic variables vs hypertension status) OR^3^ (95% CI)
Knowledge of hypertension			
Less knowledge	1		1
Intermediate knowledge	1.424 (0.779–2.604)		1.11 (0.87–1.75)
Good knowledge	1.923 (0.987–3.747)		**1.43 (1.23–3.12)**^**a**^
Smoking cigarettes/vape			
No		1	1
Yes		1.23 (0.94–2.87)	1.15 (0.87–2.13)
Consuming alcohol			
2–3 times per week		1	1
Every day		1.09 (0.81–1.35)	1.39 (0.93–1.98)
Not at all		**0.65 (0.58–0.76)**^**a**^	**0.73 (0.67–0.89)**^**a**^
Occasionally/not		**0.76 (0.67–0.98)**^**a**^	0.86 (0.77–1.16)
Once per week		0.91 (0.84–1.07)	1.01 (0.94–1.17)
The average number of days you exercise per week			
0–1		1	1
2–3		1.01 (0.89–1.11)	1.06 (0.94–1.19)
4–5		0.90 (0.84–0.97)*	0.98 (0.88–1.05)
6–7		0.68 (0.59–0,83)*	**0.83 (0.71–0.94)**^**a**^
Your ability to manage stress			
Poor		1	1
Fair		0.92 (0.73 – 1.17)	0.97 (0.78 – 1.22)
Average		0.88 (0.71 – 1.10)	0.93 (0.73 – 1.18)
Good		0.81 (0.70 – 1.02)	0.88 (0.71 – 1.09)
Very good/excellent		0.67 (0.59 – 0.94)	0.87 (0.69 – 1.09)
Highest level of education			
Primary school/some high school			1
Completed grade 12 (high school)			1.59 (0.88–2.71)
Tertiary degree			1.64 (0.68–3.16)
Current employment status			
Unemployed			1
Studying			0.98 (0.91–2.17)
Employed part-time			0.85 (0.76–1.03)
Employed full-time			**0.74 (0.69–0.80)**^**a**^
Wealth index			
1			1
2			1.01 (0.92–2.11)
3			0.91 (0.78–1.02)
4			**0.70 (0.59–0.97)**^**a**^
5			**0.65 (0.48–0.81)**^**a**^
Your quality of sleep			
Poor			1
Fair			1.14 (0.94 – 1.40)
Average			1.11 (0.89 – 1.38)
Good			1.00 (0.70 – 1.42)
Very good/excellent			0.85 (0.63 – 1.15)
How often is your blood pressure checked?			
Never			1
Monthly			**2.03 (1.78–3.23)**^**a**^
Quarterly			1.31 (0.99–2.03)
Bi-annually			1.09 (0.93–1.32)
Annually			0.98 (0.86–1.15)
Known side effects of uncontrolled blood pressure			
No			1
Yes			**1.28 (1.07–1.89)**^**a**^
Do not know			1.08 (0.91–1.63)
Known hypertension			
Biological sibling (brother/sister)			1
Biological mother			**1.79 (1.53–2.21)**^**a**^
Biological father			0.66 (0.37–1.32)
Biological grandparent			1.15 (0.88–1.37)

^a^This is a significant result.

When combining all variables, model 3 showed a significant protective association of about 26.0% between employed full-time and being hypertensive when compared to those who were unemployed. There was no significant association between education level and being hypertensive, although those with higher levels of education were likely to report not being hypertensive (Table 3).

Those who did not consume alcohol at all were 27.0% less likely to report hypertension when compared to those who consumed alcohol two or three times per week. Although drinking less alcohol as compared to regularly drinking seemed to be linked with not being hypertensive, this was not significant. Smoking seemed to be associated with being hypertensive although this was not significant.

Having a higher SES, as measured by the wealthy index (wealth index 4 and 5), was protective against hypertension when compared to those considered poor, classified as wealth index 1. Furthermore, those who exercised 6 or 7 days per week were about 17.0% likely not to be hypertensive when compared to those who did not exercise at all or just 1 day. The associations between managing stress and quality of sleep, and being hypertensive, were statistically insignificant.

There was a positive association between those with good knowledge of hypertension and being hypertensive. Those with good knowledge of hypertension were 43.0% more likely to report hypertension as compared to those with low knowledge of hypertension. This positive trend was also observed in those who had a monthly check-up of BP, who were twice as likely to be hypertensive when compared to those who had never had their BP checked. Also, knowing the side effects of uncontrolled BP and having a biological mother with hypertension was significantly associated with being hypertensive, when compared to those who did not know the side effects of uncontrolled BP as well as having a father or sibling, or grandparent with hypertension.

### Preferred strategies to prevent, reduce, or manage hypertension

**Fig. 2:**
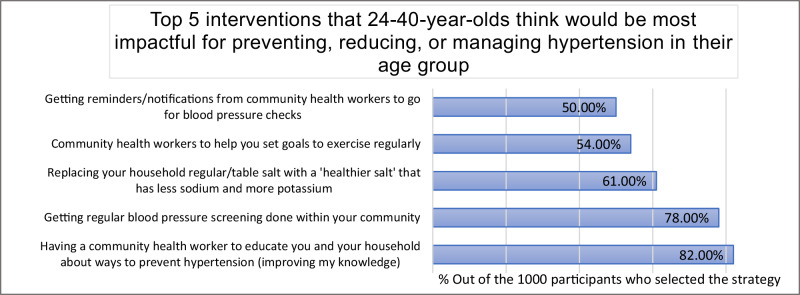
Top 5 interventions that might have an impact on preventing, reducing, or managing hypertension.

Figure 2 shows the top 5 interventions (strategies) that the respondents thought would be impactful in preventing, reducing, or managing hypertension. The most selected intervention at 82.0% was the advocation for having a community health worker to educate the respondents and their households about ways to prevent hypertension, as well as improve their knowledge.

The other options included getting regular BP screening within communities (78.0%), using ‘healthier salt’ that has less sodium and more potassium (61.0%), having community health workers help with setting goals to exercise regularly (54.0%) and getting reminders to go for regular BP checks (50.0%). There were no significant differences in the selected strategies between women and men.

## Discussion

Among our study respondents between the ages of 24 and 40 years, the reported prevalence of hypertension was high with significant differences between women and men. The prevalence of hypertension among women was 27.5% as compared to 20.4% among men. This study highlights the positive association between knowledge of hypertension, monthly BP monitoring, knowing the side effects of uncontrolled BP and having a biological mother with hypertension, and being hypertensive. Being employed full-time, having a higher SES (wealth index 4 and 5), exercising 6–7 days per week, and not consuming alcohol at all, were all found to be protective against hypertension. There were no significant associations found between the level of education, managing stress, and quality of sleep, with being hypertensive.

Although the reported hypertension prevalence in our study is slightly less than the prevalence reported in previous studies [2], what our findings show is a concerning prevalence of hypertension among young adults which indicates a high unmet need for care. The exact prevalence of hypertension in SA is not well reported but is estimated to be between 19.0% and 56.0% [25], with uncontrolled hypertension estimated higher at 13.5–75.5% [26]. Several studies in SA have shown a link between increased hypertension prevalence with increased age [27, 28], and this might explain the relatively low hypertension prevalence (24.0%) reported in this study due to the inclusion of young adults. When stratified by sex, a previous study dovetail with our findings when it reported that hypertension prevalence among people 15 years and older in SA, was higher in women at 48.0% as compared to men at 45.0% [29]. However, the South African National Health and Nutrition Examination Survey (SANHANES) 2012 and the South African Demographic and Health Survey (DHS) 2016, indicated that hypertension was significantly higher among men as compared to women, with issues such as urbanisation, high cholesterol, smoking, and alcohol consumption contributing to such differences [30, 31].

Regardless of the differences in hypertension prevalence among women and men, the reported prevalence in our study is high and a public health concern because the knowledge of hypertension and its prevention is relatively low among young adults. Furthermore, the majority of those who had good knowledge were likely to be hypertensive which indicates that they might have gotten such knowledge from having hypertension as well as interacting with health care providers. This association was reported in Northwest Ethiopia when they found that patients who had received more than 4 years of treatment had twice the odds of good knowledge about hypertension as compared to those with below 2 years of treatment [32]. The fact that those with knowledge of hypertension reported being hypertensive might point to the likelihood of good control of the condition because a study in Pakistan found an association between good knowledge of hypertension with better control of hypertension [33]. This is further supported by our study which indicated that those who are hypertensive know the negative effects of uncontrolled hypertension and this might likely mean by knowing the negative effects, those with hypertension are duly managing their condition well. This is also highlighted by the positive association that exists between having monthly BP check-ups and being hypertensive. In addition, our study showed that those with a tertiary level education were almost twice as likely to be hypertensive when compared to those with just high school even when combined with knowledge of hypertension. This might indicate that further research is needed to explore and analyse the intricate association of education attained knowledge of hypertension, and being hypertensive.

Those with a biological mother with hypertension were 79.0% likely to be hypertensive in our study and this finding is supported by previous studies that have shown a link between genetics and hypertension [34, 35]. The fact that our study has shown that women are more likely to have biological mothers with hypertension highlights the higher prevalence of hypertension among women than men, which further cements the genetic link of hypertension.

Modifiable risk factors such as physical inactivity and excessive consumption of alcohol, have been reported by World Health Organisation (WHO) as risk factors for hypertension [34]. Therefore, the findings of our study that physical activity for 6–7 days per week and not consuming alcohol at all are both protective against hypertension and are important as potential indicators for preventing and controlling hypertension. Several studies have indicated the importance of physical activity as a preventative measure against hypertension [36, 37] and this should continue to be supported and promoted to help curb the rising prevalence of hypertension. In addition, encouraging young adults to not consume or drink less alcohol might be important to prevent and reduce hypertension as indicated by our findings. Our findings further elaborate on what young adults believe are interventions to prevent, reduce, or manage hypertension among their age group. Young adults identified community health workers as a vital component in preventing, reducing, and managing hypertension as they indicated that these cadres might help educate them about ways to prevent hypertension, set physical activity goals, and remind them to regularly check for BP as well as improve their hypertension knowledge. The advocation for the use of community health workers is in line with the Pan-African Society of Cardiology (PASCAR) 10-point action plan for African ministries of health to achieve 25% control of hypertension in Africa by the year 2025 [38]. The PASCAR point 6 encourages the promotion of a task-sharing approach with adequately trained community health workers (shift paradigm) to improve knowledge, screening, identification, treatment, and control [38]. The use of a ‘healthier salt’ that has less sodium and more potassium as a strategy to help with hypertension has also been supported in previous research [39] and should be encouraged among young adults. Although not surprising, the finding that 'Community health worker to help you set goals and solutions to maintaining a healthy weight' was one of the least preferred strategies is worrying and highlights a persisting socio-cultural belief within the African community, where weight gain is perceived as a sign of living a happy and prosperous life or being attractive/desirable and fertile (for young girls and women), whereas being thin or slim is attributed to being unhealthy and suffering from HIV [40–42]. Addressing these beliefs should be integrated into health promotion and awareness initiatives to help curb overweight and obesity, especially in strategies targeting young girls and women.

Being employed full-time and having a higher SES (wealth index 4 and 5) are both associated with not being hypertensive. A study here in SA indicated that black women without medical insurance (which was linked to being poor and unemployed) were associated with being hypertensive [22]. Upward mobility in SES was found to be associated with fewer chances of being hypertensive [22], further highlighting the fact that those who are better off economically, are likely to have the tools and knowledge to prevent hypertension. Those who are in full-time employment are likely to have and can afford medical insurance, eat healthily, or seek general health check-ups hence not being hypertensive.

Although the mechanisms linking mental health conditions are not fully understood, several biological processes such as adiposity, insulin resistance, vascular function and oxidative stress, and inflammation have been implicated in the association between mental health conditions and hypertension [43]. It is proposed that chronic exposure to stress may increase the hypothalamic pituitary adrenal (HPA) axis, 'stress response system’, resulting in alterations in glucocorticoids. Glucocorticoids have also been associated with elevated waist circumference, fasting glucose, blood pressure and the presence of dyslipidaemia [44]. Exposure to stress has also been associated with greater endothelial dysfunction and increased production of pro-inflammatory cytokines, which are implicated in arterial stiffness [8, 9, 11]. Additionally, chronic stress may also reduce physical activity or result in unhealthy dietary intake, including foods with high salt intake [43].

### Strength and limitations

This study has some strengths. Since we selected participants from the nine provinces, the results of this study might be generalisable (though with caution) to the specified 24–40-year-old population in SA with internet access. Within a South African context, having internet access may be linked to a high SES and presumably better health literacy (e.g. good hypertension knowledge) and more access to healthcare services. Therefore, it is possible that the reported hypertension prevalence (24%) in the current sample might be an underestimation of the actual hypertension prevalence in the entire 24–40-year-old age group or the general population. Thus, a nationally representative survey with measured BP levels or medication use is required to accurately assess hypertension in the South African population. Secondly, the use of an adjusted odds ratio has strengthened the observed reported associations. However, there are several limitations to the current study. Firstly, we did not measure blood pressure levels for the diagnosis of hypertension or collect information on the use of antihypertensive medication. This may have led to an inaccurate estimation of the prevalence of hypertension in this sample. Secondly, there is the likelihood of our results being prone to recall bias because the outcome measure and independent variables were self-reported. Thirdly, the use of a cross-sectional design means the temporal relationship between associated risk factors and self-reported hypertension cannot be established. Since all the reported diagnoses are self-reported, there is a chance of under- or over-reporting, therefore the results should be interpreted with caution.

## Conclusion

Our study adds to the limited body of evidence concerning the prevalence of hypertension, risk factors, and related knowledge among 24–40-year-old South African men and women. The high hypertension prevalence, lack of hypertension knowledge, and reported risk factors among this age group warrant effective public health preventative measures to offset later-life CVD. Young adults identified community health workers as an important cadre to help with preventing, reducing, and managing hypertension among their age group. Furthermore, a healthier lifestyle should be encouraged and promoted among young adults, although recognised to be difficult to achieve.

## Summary table

### What is known about the topic

- Blood pressure levels increase between 30–60 years of age, which suggests that the implementation of preventative strategies during this window period may assist in combating the high and increasing prevalence of hypertension
- The increase in blood pressure levels or in the prevalence of hypertension with age especially above 50 years has been mostly associated with structural changes in large artery stiffness, which subsequently leads to high pulse pressure and pulse wave velocity.

### What this study adds

- The prevalence of hypertension, possible risk factors, and related hypertension knowledge among 24–40-year-old South Africans
- There is high prevalence of hypertension, lack of hypertension knowledge, and reported risk factors among the 24–40-year-old South Africans and this highlights the need for early robust preventative strategies to mitigate hypertension risk among this population.

## Supplementary information

Study questionnaire

## Data Availability

The datasets used and/or analysed during the current study are available from the corresponding author upon reasonable request.
