# Supplementary material for: Self-reported hypertension prevalence, risk factors, and knowledge among South Africans aged 24 to 40 years old
Source: J Hum Hypertens. 2025 Feb 24;39(2):177–87. doi: 10.1038/s41371-024-00957-8 (PMC11867972; doi:10.1038/s41371-024-00957-8)
Supplement: Supplementary file 1 — Study questionnaire [file 41371_2024_957_MOESM1_ESM.docx]

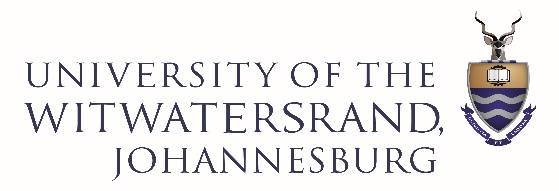

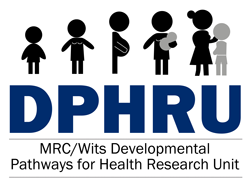

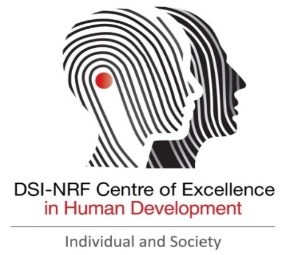


**Fast Facts Survey 2022**

**(Hypertension prevention)**

MTINTSILANA A; MAPANGA W; CRAIG A; DLAMINI S; NORRIS SA

| **QUESTION** | | **RESPONSE** |
| --- | --- | --- |
| **Basic demographic and socio-economic characteristics** | | |
| 1. | Race | 1 – White  2 – Black  3 – Indian/Asian  4 – Other |
| 2. | Marital status | 1 – Single  2 – Married/living together  3 – Separated/divorced/widowed |
| 3. | Education attained | 1 – No education  2 – Primary school /some high school  3 – Completed Grade 12 (high school)  4 – Tertiary degree |
| 4. | Employment status | 1 – Not employed  2 – Currently employed  3 – Studying |
| 5. | Please indicate the number of people living in your household or have stayed most nights in your home over the past 3 months)? | _____________ [number] |
| 6. | Please indicate if your household has the following items: | |
| 6.1 | Cellphone/Smart phone | 0 – No  1 – Yes |
| 6.2 | Computer/Tablet | 0 – No  1 – Yes |
| 6.3 | TV | 0 – No  1 – Yes |
| 6.4 | Refrigerator | 0 – No  1 – Yes |
| 6.5 | Car | 0 – No  1 – Yes |
| 6.6 | Washing machine | 0 – No  1 – Yes |
| 6.7 | Microwave | 0 – No  1 – Yes |
| 6.8 | Flush toilet inside house | 0 – No  1 – Yes |
| 6.9 | Tap water in house | 0 – No  1 – Yes |
| 6.10 | Electricity | 0 – No  1 – Yes |
| 6.11 | Generator | 0 – No  1 – Yes |
| 6.12 | Air conditioner | 0 – No  1 – Yes |
| **Medical-related information** | | |
| 7. | Do you know what the side-effects are of having uncontrolled high blood pressure? | 0 – No  1 – Yes  2 – Don’t know |
| 8.1 | How often do you get your blood pressure checked? | 1 – Monthly  2 – Quarterly  3 – Bi-annually  4 – Annually  5 – Never |
| 8.2 | If never, is this a result of the following: | 1 – Lack of transport to a health facility  2 – Your health care facility does not offer this procedure  3 – BP checking is not important |
| 9. | Has a doctor or nurse or health worker at a clinic or at hospital told you that you had or have any of the following conditions: | |
| 9.1 | Hypertension or high Blood Pressure | 0 – No  1 – Yes  2 – Don’t know |
| 9.2 | High blood cholesterol or fats in the blood | 0 – No  1 – Yes  2 – Don’t know |
| 9.3 | Diabetes | 0 – No  1 – Yes  2 – Don’t know |
| 9.4 | Overweight or obesity | 0 – No  1 – Yes  2 – Don’t know |
| 9.5 | Chronic kidney disease | 0 – No  1 – Yes  2 – Don’t know |
| **Lifestyle factors** | | |
| 6.1 | Do you smoke? | 0 – No  1 – Yes |
| 6.2 | If yes, how many do you smoke a day?  *(This may include but not limited to, cigarettes with a filter or rolled, chewing tobacco, pipe, cigars, vape, hubbly, snuff or dagga)* | 1 – < 1/day  2 – 1-5/day  3 – 6-10/day  4 – 11-20/day  5 – > 20/day |
| 7.1 | Do you consume alcohol? | 0 – No  1 – Yes |
| 7.2 | If yes, how many times during the week do you consume alcohol? | 1 – occasionally / not every week  2 – once/week  3 – 2-3 times/week  4 – every day |
| 8. | How many days on average in a week do you exercise for more than 30minutes a day hard enough to increase your heart rate and for you to break a sweat? | ___________ [days] |
| **Familial history** | | |
| 9. | Do/did any of your blood relatives ever have any of the following conditions: you may choose more than one option if applicable | |
| 9.1 | Hypertension / high blood pressure | 0 - No  1 - Biological mother  2 - Biological father  3 - Biological grandparent  4 - Biological sibling (brother/sister)  5 - Biological pibling (aunt/uncle)  6 - Biological child (offspring)  7 - Don’t know |
| 9.2 | Diabetes | 0 - No  1 - Biological mother  2 - Biological father  3 - Biological grandparent  4 - Biological sibling (brother/sister)  5 - Biological pibling (aunt/uncle)  6 - Biological child (offspring)  7 - Don’t know |
| 9.3 | Overweight or obesity | 0 - No  1 - Biological mother  2 - Biological father  3 - Biological grandparent  4 - Biological sibling (brother/sister)  5 - Biological pibling (aunt/uncle)  6 - Biological child (offspring)  7 - Don’t know |
| 9.4 | Heart disease or heart attack | 0 - No  1 - Biological mother  2 - Biological father  3 - Biological grandparent  4 - Biological sibling (brother/sister)  5 - Biological pibling (aunt/uncle)  6 - Biological child (offspring)  7 - Don’t know |
| 9.5 | Stroke | 0 - No  1 - Biological mother  2 - Biological father  3 - Biological grandparent  4 - Biological sibling (brother/sister)  5 - Biological pibling (aunt/uncle)  6 - Biological child (offspring)  7 - Don’t know |
| 9.6 | High cholesterol | 0 - No  1 - Biological mother  2 - Biological father  3 - Biological grandparent  4 - Biological sibling (brother/sister)  5 - Biological pibling (aunt/uncle)  6 - Biological child (offspring)  7 - Don’t know |
| 9.7 | Mental health condition or are you currently taking treatment for your mental condition (e.g., Depression, anxiety, bi-polar)? | 0 - No  1 - Biological mother  2 - Biological father  3 - Biological grandparent  4 - Biological sibling (brother/sister)  5 - Biological pibling (aunt/uncle)  6 - Biological child (offspring)  7 - Don’t know |
| **We will now ask you some questions around what you know of hypertension**  **(An adapted hypertension evaluation of lifestyle and management (HELM) questionnaire**) | | |
| 10. | If someone’s blood pressure is 115/75 mmHg, it is … | 1 – High  2 – Low  3 – Normal  4 – Don’t know |
| 11. | If someone’s blood pressure is 160/100 mmHg, it is … | 1 – High  2 – Low  3 – Normal  4 – Don’t know |
| 12. | Once someone has high blood pressure, it usually lasts for … | 1 – A few years  2 – 5-10yrs  3 – Rest of their life  4 – Don’t know |
| 13. | People with high blood pressure should take their medicine … | 1 – Everyday  2 – Few times a week  3 – Only when they feel sick  4 – Don’t know |
| 14. | Losing weight usually makes blood pressure … | 1 – Go up  2 – Go down  3 – Stay the same  4 – Don’t know |
| 15. | Eating less salt usually makes blood pressure … | 1 – Go up  2 – Go down  3 – Stay the same  4 – Don’t know |
| 16. | High blood pressure can cause heart attacks, if left untreated. | 0 – No  1 – Yes  2 – Don’t know |
| 17. | High blood pressure can cause kidney problems, if left untreated. | 0 – No  1 – Yes  2 – Don’t know |
| 18. | High blood pressure can cause diabetes, if left untreated. | 0 – No  1 – Yes  2 – Don’t know |
| 19 | High blood pressure can cause a person to have a stoke, if left untreated. | 0 – No  1 – Yes  2 – Don’t know |
| 20. | Moderate-to-vigorous exercise (30min/day) 3-5 times per week lowers blood pressure. | 0 – No  1 – Yes  2 – Don’t know |
| 21. | Smoking a pack of cigarettes per day will not affect a person’s risk for hypertension. | 0 – No  1 – Yes  2 – Don’t know |
| 22. | Motivational interviewing techniques are not useful when guiding a person to make lifestyle changes. | 0 – No  1 – Yes  2 – Don’t know |
| 23. | High blood pressure cannot be cured. | 0 – No  1 – Yes  2 – Don’t know |
| 24. | A hypertensive individual should strive for a normal blood pressure of 120/80 mmHg. | 0 – No  1 – Yes  2 – Don’t know |
| 25. | A person with high blood pressure should eat less fat. | 0 – No  1 – Yes  2 – Don’t know |
| 26. | A person with high blood pressure should eat more fruit and vegetables. | 0 – No  1 – Yes  2 – Don’t know |
| 27. | The best type of meat for people with high blood pressure is white meat. | 0 – No  1 – Yes  2 – Don’t know |
| 28. | The best type of meat for people with high blood pressure is red meat. | 0 – No  1 – Yes  2 – Don’t know |
| 29. | The best cooking method of people with high blood pressure is frying. | 0 – No  1 – Yes  2 – Don’t know |
| 30. | The best cooking method of people with high blood pressure is boiling or grilling. | 0 – No  1 – Yes  2 – Don’t know |
| 31. | Do you know what low-sodium salt is? | 0 – No  1 – Yes  2 – Don’t know |
| 32. | Rate your confidence in detecting and treating high blood pressure. | 1 – Very confident  2 – Confident  3 – Not so confident / need more guidance |
| **We would now like to know your perceptions and recommendations about the prevention, reduction, and management of hypertension** | | |
| 33.. | Select the **top 5 interventions** that you think would be most impactful for preventing, reducing, or managing hypertension (or high blood pressure) in your age group and community:   - Having a community health worker to educate you and your household about ways to prevent getting hypertension (improving my knowledge) - Getting regular blood pressure screening in your community - Getting reminders/notification from the community health worker to go for your blood pressure measurement and to take my medication regularly if diagnosed with hypertension - Community health worker to help you set goals and solutions to exercise regularly - Community health worker to help you set goals and solutions to maintaining a healthy weight - Replacing your household regular/table salt with a “healthier salt” that has less sodium and more potassium - Getting good sleep quality - Community health worker helping to managing stress levels better - Quitting smoking | |
| 34. | If a community health worker provides you with information and resources to live a healthy lifestyle so that you do not develop hypertension and other chronic disorders (diabetes, stroke, and heart attack) when you are older, how likely are you to use this information? | 0 – Very likely  1 - Likely  2 - Neutral  3 – Not likely  4 – Very unlikely  5 – Don’t know |
| 35. | If a community health worker offers to measure your blood pressure levels, weight, and other important health measurements for free at home, how likely are you to use this service? | 0 – Very likely  1 - Likely  2 - Neutral  3 – Not likely  4 – Very unlikely  5 – Don’t know |
| 36. | If you were encouraged to exercise for more than 30minutes a day hard enough to increase your heart rate and for you to break a sweat, how many days in a week would you exercise? | 0 – Zero (0) days per week  1 – Once per week  2 – 2-3 times per week  3 – 5 days per week  4 – Every day of the week  5 – Don’t know |
| 37 | Would you change your diet for a period of 3-6 months if you needed to lose weight to reduce your risk of hypertension? | 0 – No  1 - Yes  2 – Don’t know |
| 38. | If a community health worker encourages you to stop or reduce your smoking or alcohol intake to reduce your risk of hypertension, how likely are you to do it? | 0 – Very likely  1 - Likely  2 - Neutral  3 – Not likely  4 – Very unlikely  5 – Don’t know |
| 39. | Would you like to receive nutritional information from a professional (dietician) if available? | 0 – No  1 - Yes  2 – Don’t know |
| 40. | Do you add salt to food at the table? | 0 – Always  1 - Rarely  2 - Sometimes  3 – Not likely  4 – Often  5 – never |
| 41. | From your perspective, how much salt do you think you consume? | 0 – Far too much  1 – Too much  2 – Just the right amount  3 – Too little  4 – Far too little  5 – Don’t know |
| 42. | If you and your household were asked to use “a healthier salt” that tastes like ordinary salt instead of the regular salt, how likely are you use it? | 0 – Very likely  1 - Likely  2 - Neutral  3 – Not likely  4 – Very unlikely  5 – Don’t know |
| 43. | If you were given low-sodium salt for free which of the following applies? | 1 - I would be happy to try it until it was finished  2 - I would try it but stop using it if it tasted different  3 - I would not trust it and would use my regular salt instead  4 - I would use it for some things and use my regular salt for other things |
| 44. | If you said you would not trust the low sodium salt, would it change your mind if someone explained what was different about it? | 0 – No  1 - Yes  2 – Don’t know |
| 45. | During the past month, how would you rate your sleeping quality overall? | 0 – Poor  1 - Fair  2 - Average  3 – Good  4 – Very good/excellent  5 – Don’t know |
| 46. | How would you rate your ability to manage stress? | 0 – Poor  1 - Fair  2 - Average  3 – Good  4 – Very good/excellent  5 – Don’t know |
| 47. | Would you like to receive information or training on how to manage your stress if it were available in your community? | 0 – No  1 - Yes  2 – Don’t know |
| 48. | Do you or would you use traditional or herbal medicines to treat or control your hypertension? | 0 – No  1 – Yes  2 – Don’t know |
| 49. | If you need more knowledge on high blood pressure (hypertension) where would you get it | 0 - Local/community clinic nurse  1 - Public hospital  2 - Private doctor  3 - Asking my family or friends who are hypertensive  4 - Don’t know  5 - Other  If Other, specify _________________ |
| 50. | If someone is diagnosed with hypertension, do you think it is important that they control it? | 0 – Strong agree  1 – Agree  2 – Neutral  3 – Disagree  4 – Strongly disagree |
